# Intervening on Conflict, Parental Bonds, and Sexual Risk Acts among Adolescent Children of Mothers Living with HIV

**DOI:** 10.1371/journal.pone.0101874

**Published:** 2014-07-10

**Authors:** Mary Jane Rotheram-Borus, Judith A. Stein, Eric Rice

**Affiliations:** 1 Department of Psychology, University of California Los Angeles, Los Angeles, California, United States of America; 2 School of Social Work, University of Southern California, Los Angeles, California, United States of America; University of Cape Town, South Africa

## Abstract

**Objective:**

In 1993–1994, a psychosocial intervention conducted in New York City significantly improved outcomes for parents living with HIV and their adolescent children over six years. We examine if the intervention benefits are similar for adolescents of mothers living with HIV (MLH) in 2004–2005 in Los Angeles when MLH’s survival had increased substantially.

**Methods:**

Adolescents of MLH in Los Angeles (N = 256) aged 12–20 years old were randomized with their MLH to either: 1) a standard care condition (n = 120 adolescent-MLH dyads); or 2) an intervention condition consisting of small group activities to build coping skills (n = 136 adolescent-MLH dyads, 78% attended the intervention). At 18 months, 94.7% of adolescents were reassessed. Longitudinal structural equation modeling examined if intervention participation impacted adolescents’ relationships with parents and their sexual risk behaviors.

**Results:**

Compared to the standard care, adolescents in the intervention condition reported significantly more positive family bonds 18 months later. Greater participation by MLH predicted fewer family conflicts, and was indirectly associated with less adolescent sexual risk behavior at the 18 month follow-up assessment. Anticipated developmental patterns were observed - sexual risk acts increased with age. Reports were also consistent with anticipated gender roles; girls reported better bonds with their mothers at 18 months, compared to boys.

**Conclusions:**

Adolescents of MLH have better bonds with their mothers as a function of participating in a coping skills intervention and reduced sexual risk-taking as a function of MLH intervention involvement.

## Introduction

HIV has been transformed from a terminal to a chronic disease in the last 15 years [1]. Concurrently, the types of challenges facing parents diagnosed with HIV have changed dramatically, especially those involving their children [2]. In the 1990s, when an HIV diagnosis meant a premature death, we developed Project TALC (Teens and Adults Learning to Communicate) an efficacious intervention to improve health outcomes for both parents and adolescents, as well as to enhance the quality of their relationships [3], [4]. In view of the change in HIV-positivity into a chronic illness, we adapted the previously efficacious intervention to account for new challenges, to see if similar benefits were found. The impact of the adapted intervention was far less than when parents with HIV faced a terminal illness [2]. This paper examines the factors mediating the impact of the adapted intervention, specifically adolescents’ family relationships and sexual risk.

Parents’ terminal illnesses significantly increase their children’s stress, depression, and comportment, especially among adolescents [3], [5]. In the early 1990s, a parent diagnosed with AIDS could expect to live about 15 months [6]. There were no clear treatments that could prolong life, and the treatments had many side effects [7]. In this context, parents were challenged to prepare themselves and their children for their death. Their children needed to grieve and prepare themselves for a home with new caretakers. Parents with AIDS were challenged not only by their health status, but by being marginalized by their risk histories, socioeconomic status, and ethnicity. In New York City in 1994–1995, 87% of parents with AIDS were current or former injecting drug users, disenfranchised (typically receiving child welfare and housing benefits), and African American or Latino background [8].

To help parents with AIDS, we designed Project TALC, a 24 session intervention delivered in three modules, timed to the phase of parental illness. In the first module, parents met in small groups without their children to emotionally adapt to their HIV diagnosis, especially coming to terms with their potential premature death and deciding who, how, what, and when to disclose their HIV status. Almost all parents disclosed to their adolescent children. In the second module, parents and adolescents met in small groups in order to concurrently support the mother to cope effectively with her symptoms of HIV illness and to improve communication, reduce conflict, and set healthy daily routines for the family. The third module was planned to support adolescents in their post-death adjustment, when the adolescent had a new caregiver. Unexpectedly, almost half of the parents with AIDS were alive six years after diagnosis, due to substantial improvements in the quality of care [4] and the introduction of antiretroviral (ARV) medications [9]. The need for a post-death intervention module is not relevant today for children of parents living with HIV.

Being randomized to receive the Project TALC intervention led to substantial benefits for both the parents with AIDS and the adolescent, benefits which persisted for six years following the intervention delivery. Intervention parents were more likely to stop abusing drugs and became less depressed than those in the control condition [3]. Both parental drug use and depression have been repeatedly linked to poor child outcomes [10], [11] and these reductions were associated with benefits to their adolescent children [3], [4]. The adolescents in the intervention condition were less likely to use alcohol and/or drugs, delayed the onset of sexual debut, and engaged in less unprotected sex [3]. The adolescents had fewer sexual partners, fewer babies, and their babies were more likely to have better home environments and tended to have higher IQ over the next six years [3], [4], [12]–[18]. These results are impressive. However, these benefits were observed among a sample of parents and adolescents experiencing extreme stress.

In this original Project TALC intervention, fathers with AIDS were included. However, almost no fathers actually lived with their adolescent children [3]. Therefore, in designing intervention 10 years later, we focused on mothers living with HIV (MLH).

Today, the challenges faced by MLH have shifted dramatically, especially because of the widespread dissemination of effective anti-retroviral therapy (ARV) in the United States [9], [19]. Rather than acute stress of potential death, ongoing hassles characterize the lives of parents with HIV, and stress can realistically be expected to be lifelong [19]. MLH continue to need to cope and adapt to their HIV status, adhere to medical regimens, and parent well while living with HIV [20]–[22]. The potential benefits of ARV treatments have become much greater, but the medical regimens are much more complex than in the 1990s and require lifelong adherence [9]. Concurrently, the risk profiles of MLH have changed dramatically in the last 10 years.

This is particularly likely when focusing on MLH in Los Angeles. Similar to New York City, MLH in Los Angeles are likely to be African-American or Latino [23]. However, the African-American MLH in New York City had been injecting drug users [24], and most Latina and African-American MLH in Los Angeles become HIV infected because of their sexual partner’s risk, and living in one of the sixneighborhoods with high seroprevalence rates [23]. Geography, rather than individual risk acts, is associated with HIV infection in Los Angeles. Latina MLH are often married Mexican or Central American immigrants, who may have immigrated separately from their spouses with an intervening period of several years [23]. African American women are partnered with injecting drug users (but are not themselves drug users) or have partners who have been jailed or who are behaviorally bisexual, without the woman’s knowledge [23]. Thus, compared to 10 years earlier, there are significant and dramatic differences in risk histories, disease trajectories, and neighborhood clustering in HIV infection in 2004–2005.

To address different challenges associated with chronic HIV and to serve mothers with less of a personal sexual and substance use risk history, we adapted the Project TALC intervention [25]. We reduced the number of intervention sessions and changed the focus from specific, imminent health challenges to a focus on enhancing long-term coping with illness, parenting skills and encouraging achievement of adolescents’ healthy developmental milestones. Thus, rather than focusing on a parent/child population whose history indicates a need for intervention, we delivered a preventive intervention for both MLH and adolescents.

Bonds with parents have important long-term benefits, including the ability to cope with stressors and challenges [29]. Strong parental bonds are protective to children, supporting successful completion of developmental tasks in adolescence [30], [31]. Positive parent-child connectedness is generally protective against emotional distress, violence, and problem behaviors, such as substance use and sexual behavior [32], [33]. Families coping with HIV tend to report having stronger bonds with their adolescent children as compared to a family where there is not a MLH [27]. However, because HIV is a chronic disease, the challenges facing MLH may rise as the HIV disease impacts daily life, weakening bonds over the long term. MLH’s stressors related to living with HIV are likely to impact their bonds with their children.

In our previous randomized controlled trial [RCT], adolescent children of parents living with HIV often become parentified, that is, the adolescents assumed the parents’ roles when the MLH was too ill to take care of the family [26]–[28]. In these circumstances, family conflict was high [17] and the Project TALC reduced family conflict. Given these earlier findings, we anticipated that addressing parenting in the intervention would be likely to reduce family conflict.

In addition to family conflict created by parentification, other by-products of stress in the household include weakened parent-child relationships, and risky sex and substance use by adolescents [34]. Adolescent children of MLH show higher levels of problematic substance use and psychological distress compared to adolescents living in the same neighborhoods in Los Angeles [2], [35]. The current study hypothesizes that MLH’s involvement in Project TALC may be protective in encouraging their children to have lower levels of sexual risk, lessened conflict and stronger maternal bonds.

Of particular importance to understanding the efficacy of interventions are the mediating features of intervention-program involvement [36]. Thus, the current study analyzes whether the involvement of MLH in the intervention mediated improvements in their children’s outcomes. Specifically, in this article we examine how maternal involvement in the Project TALC intervention impacts family conflict, parental bonds, and sexual risk behavior among the adolescents of MLH. We hypothesize that demographic characteristics, such as age, gender, extent that adolescents reported engaging in sexual acts or risky behavior, as well as quality of family bonds, mediated by assignment to a coping skills intervention and level of participation in the program. We include prior behaviors as an important control on subsequent behaviors. We did not include substance use because it was extremely infrequent in this population of adolescents.

## Methods

### Ethics Statement

All research involving human participants was reviewed and approved by the UCLA South General Campus Institutional Review Board, (study numbers #02-11-031 & #10-001354) before any human subject contact was made.

- Adult participants provided their written informed consent, which was documented by signing the UCLA-IRB reviewed and approved *Mother/Female Caregiver Informed Consent Form*.
- Adult participants also provided their written permission for their child(ren) aged 12–17 to participate in the study, which was documented by the Mother/Female Caregiver participant signing the UCLA-IRB reviewed and approved *Parent/Caregiver Permission for Youth Participation*.
- Youth who participated in the study provided their written informed assent/consent which was documented by each youth signing the following (age-appropriate) Informed Assent/Consent Forms, which were all reviewed and approved by the UCLA-IRB:
  1. 12 year old participants signed the *Youth Assent –12 Year Olds*;
  2. Youth participants aged 13–17 signed the *TALK _LA_ Youth Assent;*
  3. Youth participants aged 18–20 signed the *TALK _LA_ Youth Consent.*

### Participants

**Figure 1:**
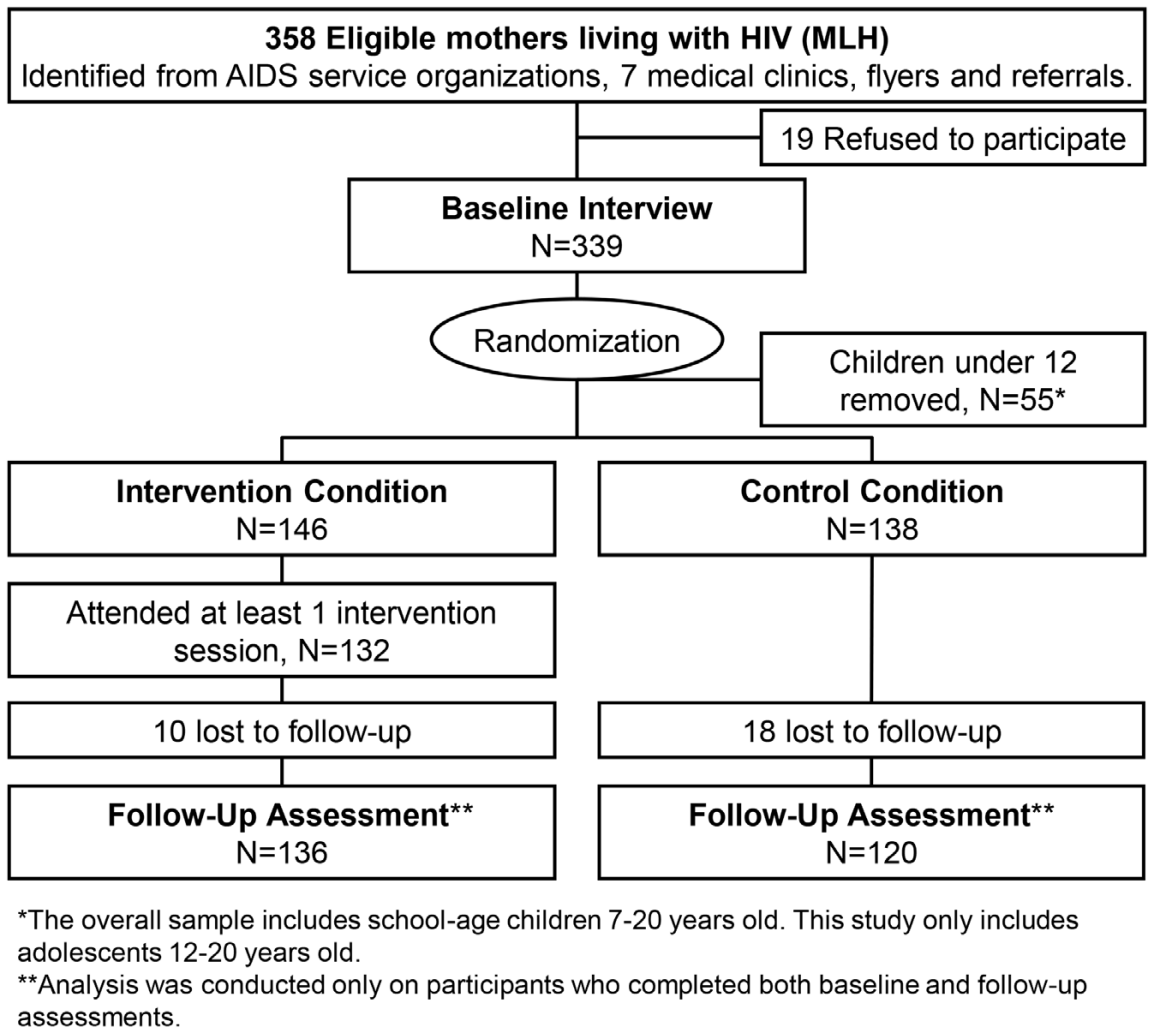
Study Randomization Design & Participant Flow among adolescents aged 12–20 years old.

IRB approval was obtained for the recruitment of MLH in Los Angeles County from AIDS Service Organizations that provided supportive social services to families (42%), seven medical clinics (53%), and via referrals and flyers (5% of sample; refusal rate, 6.4%) from January 2005 to October 2006. MLH were approached in waiting rooms of clinics, referred by providers to study staff, or approached by members of the study staff when attending events for persons living with HIV (N = 358). The inclusion criteria consisted of being diagnosed with HIV; not having a health care provider express concerns about intervention participation; being the primary residential caretaker of a school aged child from age 6–20 years old; and not to appear psychotic to an interviewer by verbalizing hallucinations or demonstrating incoherent speech. Voluntary informed consent was obtained from each MLH and her school-aged children; 94% of MLH completed the baseline interview (n = 339). Randomization was conducted immediately following the baseline interview by telephoning a central data coordinator. MLH and the children randomized to the standard care condition were offered the intervention after 18 months. Figure 1 outlines the flow of MLH and their children through the study, indicating the subsample used in the analyses, which only included adolescents aged 12–20 years old.

#### Sample used for current analyses

Only adolescent children aged 12–20 years old (N = 256) were included in the current analyses. The mean age of the adolescents at baseline was 14.7 years (SD = 2.3) and 60% were female. Among the adolescents, 70% were Hispanic, 24% were African-American, 4% were white, and 2% were of other ethnicities. The follow-up rate was 94.7% for the adolescent children. We examined any differences between adolescents successfully followed over time and those lost to follow-up; there were no significant differences.

#### Standard care

All MLH in Los Angeles have access to primary health care, specialized HIV care, antiretroviral (ARV) medications, and reimbursement for transportation. There are no waiting lists for HIV medications or services and transportation tokens are provided by clinics. Family members do not receive any services.

#### Intervention

For adolescents, the principal intervention goals were to: 1) reduce HIV risk behaviors; 2) improve parent-child relationships; and 3) reduce family conflict. For MLH, intervention goals were designed to: 1) increase adherence to medical regimens, including ARV; 2) reduce sexual and drug use transmission acts; 3) reduce mental health symptoms; and 4) improve parenting while ill (i.e., reduce family conflict, improve communication, and clarify family roles).

The intervention was delivered in either English or Spanish, to small groups of 5 to 8 MLH and their children. Groups typically met twice weekly for 1.5 to 2 hours each over eight weeks (n = 16 sessions). Both mothers and adolescents were invited to the intervention, yet not all mothers attended with their children. Attendance was high, especially if the mothers invited their children. MLH were compensated for transportation and child care costs for the first 16 sessions they attended, and adolescents were provided with $10 gifts. Overall, 97% of MLH attended at least one session and, among women attending at least one session, 81% attended 12 or more of the 16 sessions offered. Participants were welcome to attend additional sessions without incentives (45 MLH and 11 adolescents did so). We have discussed previously the successes and challenges faced in the delivery of this intervention in community settings in Los Angeles [37].

### Measures

All assessments were conducted in English or Spanish, with the adolescent selecting their language preference, by trained interviewers using laptop computers to record responses. When sensitive topics were asked (e.g., sexual risk acts and drug use), the adolescent entered their responses directly into the computer using audio-assisted computer self-interviewing software. The measures had been used previously in our study of parents with AIDS [3].

The longitudinal analyses were conducted with structural equation models (SEM) using latent variables [38]. A “latent variable” refers to a set of clustered measures that indicate a single underlying construct which provides a higher level of abstraction. We define below each of the indicators in each latent variable. Latent variables discern relationships among different constructs. The latent variables were constructed in exactly the same way at the baseline and the 18 month outcome assessment, in order to control for prior behaviors. Some predictors are single-item demographic characteristics and one is an indicator of being in the intervention condition or not (i.e., the standard care condition). All items were reported by the adolescents.

#### Background measures

Demographic predictors included the adolescent’s age in years at study entry, gender of the adolescent (1 = male, 2 = female), and whether he or she was Hispanic (scored 0 (no) or 1 (yes)). All other races/ethnicities (White, African-American, etc.) were the reference condition.

#### Measures at baseline and at 18 month outcome

Conflict, among adolescents and their mothers, was assessed with seven items from the Conflict-Resolution Behavior Questionnaire [39]. Coefficient alpha for the 7 items = .82. The scale has been validated previously for adolescents [39]. The scale, administered to the adolescents, assesses the adolescent’s style of conflict resolution with their mother in the past 6 months, as well as the frequency that style was employed. Items range from 0–6 (0 = never, 1 = once, 2 = twice, 3 = a few times, 4 = some of the time, 5 = most of the time, 6 = every time). As an example, one item states: “You tried to discuss the issue relatively calmly” and another item states: “You insulted or swore at her.” Items were coded so that high scores indicate more conflict and poorer conflict resolution skills. Items were combined at random to create three parcels to avoid too many indicators for the size of the sample. This is acceptable when coefficient alpha is high among the items [40].

The Parker Bonding Instrument (PBI) was administered to the adolescents [41]–[43]. The items were rated on a 1 to 4 scale (“strongly agree” to “strongly disagree”) and reverse scored where appropriate so that high scores indicated better bonding. The Parker Bonding Instrument has been tested in a variety of situations for test and retest reliability and has demonstrated satisfactory construct and convergent validity [42]. Research with the PBI has demonstrated associations among adolescent mental health, substance use, and parental bonds [44]–[47]. The Parental Bonding factor of caring was used in the current analysis, based on our previous work with parents with AIDS [11], [16] (4 items; coefficient alpha = .73). An example of the items includes: “She appeared to understand my problems and worries.”

Sexual Risk Behavior was self-reported by adolescents on two items: 1) the number of sex partners in the past 6 months; and 2) the percent of sexual encounters without using a condom in the past 6 months. This item was calculated by dividing the number of sexual encounters without a condom by the number of times they had sex in the past six months. Those abstaining from sex were assigned a score of 0%.

#### Intervention status and participation

Adolescents and their MLH were randomized to the Project TALC intervention (1) or to standard care condition (0). Because there was a wide variation in attendance, we also include a variable representing the number of sessions attended of Project TALC by the MLH (*amount of maternal participation*).

### Analytic Strategy

The confirmatory and predictive path analyses were performed using the EQS structural equations program software [38]. Latent variable analysis allows one to evaluate a model with directional hypotheses with correlational, non-experimental data. An initial confirmatory factor analysis (CFA) assessed the adequacy of the hypothesized measurement model and the associations among the latent variables and the single item variables. Correlations between some of the error residuals of similar items at the two time periods were allowed for fit improvement based on results of the LaGrange Multiplier Test (LM Test) [48]. This test suggests significant paths or covariances that can improve the fit of the model. Then a latent variable path model compared baseline variables to those collected at 18 months. We positioned the baseline variables of adolescent gender, age, and ethnicity (Hispanic) as predictors of the intermediate baseline measures of Conflict, Positive Parental Bonds, and Sexual Risk Behavior. These baseline measures in turn predicted adolescent reports at 18 months of Conflict, Positive Parental Bonds, and Sexual Risk Behavior. We expected stability over time between similar constructs (e.g., baseline Conflict and 18-month Conflict). Random assignment to the Intervention Condition (or not) and the amount of parental participation in the intervention were examined as further predictors of the 18 month outcomes, over and above the expected stability of the same constructs across time. Because of random assignment we did not expect intervention condition membership to be correlated with any baseline predictors. However, It was possible that amount of participation could be associated with some of the baseline variables, due to greater engagement and interest in the program by some of the mothers. We accounted for those relationships by allowing associations between participation and background predictors, if they were suggested by the LM Test and were significant. We also report whether there were significant indirect effects on 18-month outcomes of any demographic baseline predictors mediated through the baseline latent variables.

Goodness-of-fit was assessed with the robust Satorra-Bentler χ^2^ (S-B χ^2)^, the Robust Comparative Fit Index (RCFI), and the root mean squared error of approximation (RMSEA) [37], [49]. The Robust S-B χ^2^ was used because it is appropriate when the data depart from multivariate normality; the data in this study did depart from this assumption. The multivariate kurtosis estimate was high (Mardia normalized *z*-statistic = 58.71) rejecting multivariate normality. The RCFI ranges from 0 to 1 and reflects the improvement in fit of a hypothesized model over a model of complete independence among the measured variables. RCFI values at.95 or greater are desirable, indicating that the hypothesized model reproduces 95% or more of the covariation in the data. The RMSEA is a measure of lack of fit per degrees of freedom, controlling for sample size, and values less than.06 indicate a relatively good fit between the hypothesized model and the observed data.

## Results

### Confirmatory Factor Analysis

Table 1 reports summary statistics of the measured variables and the factor loadings of the hypothesized factor structure. The factor analysis confirmed the viability of the constructs hypothesized as latent variables. In addition, summary statistics are also reported separately for the intervention and standard care conditions for reader interest. However, it was not feasible to perform meaningful separate path analyses by group. The sample sizes were not large enough because of the number of parameters to be estimated in the model. All factor loadings were significant (*p*≤.001). Fit indexes for the final CFA model were excellent: S-B χ^2^ = 195.14, 178 *df*; RCFI = .97; RMSEA = .022, Confidence interval (CI) for RMSEA = .000 to.039. Two reasonable correlated error residuals were added between similar items across time, as suggested by the LM test, for fit improvement. These included the residuals between conflict resolution parcel 1 at baseline and at follow-up, and conflict resolution parcel 2 at baseline and at follow-up.

**Table 1:** Means or percentages, standard deviations, ranges, and factor loadings of measured variables in the Confirmatory Factor Analysis.

	Total Sample N = 256		Intervention N = 136		Control N = 120	
Latent and MeasuredVariables (range)	Mean (S. D.)/percentage	Factor Loading*	Mean (S. D.)/percentage	Factor Loading	Mean (S. D.)/percentage	Factor Loading
*Baseline adolescent variables*						
Gender female	60%	NA**	60%	NA	61%	NA
Age (range = 12–18 years)	14.72 (2.01)	NA	14.60 (2.25)	NA	14.85 (2.40)	NA
Hispanic	73%	NA	65%	NA	81%	NA
Conflict (0–6)						
Conflict Composite 1	1.03 (1.25)	.85	0.95 (1.20)	.87	1.12 (1.29)	.85
Conflict Composite 2	1.30 (1.38)	.85	1.27 (1.36)	.89	1.32 (1.40)	.79
Conflict Composite 3	0.27 (0.74)	.62	0.28 (0.74)	.58	0.26 (0.74)	.61
Positive Parental Bonds (1–4)						
Spoke to you with a warm voice	3.37 (1.17)	.75	3.36 (0.78)	.68	3.37 (0.78)	.79
Did not help as much as needed (***R)	3.23 (0.97)	.42	3.23 (1.04)	.35	3.22 (0.90)	.51
She understood your problems	3.25 (0.89)	.71	3.24 (0.88)	.64	3.25 (0.91)	.78
She was affectionate to you	3.37 (0.80)	.65	3.40 (0.54)	.66	3.34 (0.76)	.66
Sex Risk Behavior past 6 months						
Number of sex partners (0–6)	0.27 (0.68)	.43	.20 (0.49)	.57	0.33 (0.83)	.36
% times sex without condom(0–100%) condom	4.75%	.78	1.63%	.50	8.00%	.94
Intervention participant	51%	NA	NA	NA	NA	NA
Amount of parent participation (0–36 sessions)	7.72 (10.03)	NA	NA	NA	NA	NA
*18 month adolescent outcomes*						
Family Conflict (0–6)						
Conflict Composite 1	0.75 (1.08)	.71	0.65 (0.98)	.69	0.85 (1.17)	.79
Conflict Composite 2	0.83 (1.17)	.90	0.79 (1.17)	.95	0.86 (1.18)	.81
Conflict Composite 3	0.13 (0.47)	.48	0.14 (0.54)	.48	0.11 (0.38)	.44
Positive Parental Bonds (1–4)						
Spoke to you with a warm voice	3.11 (0.58)	.67	3.19 (0.54)	.70	3.02 (0.61)	.55
Did not help as much as needed (R***)	2.97 (0.60)	.48	3.06 (0.59)	.66	2.87 (0.60)	.28
She understand your problems	2.86 (0.62)	.40	2.94 (0.61)	.26	2.78 (0.63)	.60
She was affectionate to you	3.00 (0.55)	.68	3.06 (0.52)	.67	2.94 (0.57)	.70
Sex Risk Behavior past 6 months						
Number of sex partners (0–20)	0.66 (2.01)	.49	.53 (0.94)	.50	0.81 (2.69)	.51
% times sex without condom (0–100%)	12.22%	.68	11.22%	.83	13.30%	.66

*All factor loadings significant, p≤.001. Factor loadings are standardized.

**NA = Not applicable.

***R = Reverse-scored.

Table 2 reports the bivariate correlations among the variables in the model, before the directional hypothesized longitudinal path model was tested. Focusing on the most significant findings, there are several notable relationships among the variables. As has been reported in the literature, adolescents typically are more likely to experience sexual debut and engage in more sexual behavior with increasing age [50]. In this study, findings were similar: the older adolescents were more sexually active at the baseline assessment (r = .32) and again at the 18 month assessment (r = .38). By chance there were fewer Hispanic youth in the intervention condition and, thus, less parental participation by Hispanics due to the composition of the sample. Females were significantly more likely to report greater family conflict at both time periods (.19 and .20 respectively).

**Table 2:** Correlations among model constructs for 256 adolescents.

Variables	1	2	3	4	5	6	7	8	9	10
**Baseline**										
1. Adolescent age	__									
2. Hispanic	−.12	__								
3. Female adolescent	.07	.10	__							
4. Conflict	.01	−.05	.19**	__						
5. Positive Parental Bonds	−.01	−.07	−.11	−.45***	__					
6. Sexual Risk Behavior	.32***	.13	.19*	.18*	−.02	__				
**18-Month Outcomes**										
7. Conflict	−.13*	.03	.20**	.53***	−.17*	.03	__			
8. Positive Parental Bonds	.09	.04	.11	−.39***	.51***	.09	−.06	__		
9. Sexual Risk Behavior	.38***	−.09	.12	−.04	.20*	.68***	−.06	.04	__	
**Intervention variables**										
10. Intervention condition member	−.05	−.18**	−.01	−.04	.01	−.21**	−.04	.23**	−.07	__
11. Amount of parent participation	−.01	−.14*	−.04	−.09	−.12	−.23**	−.15*	.08	−.14*	.73***

* = p≤.05, ** = p≤.01, *** = p≤.001.

Positive Parental Bonds were negatively associated with Conflict at baseline (−.45). Similar constructs showed substantial stability across time (.53 for Conflict,.51 for Positive Parental Bonds, and .68 for Sexual Risk Behavior). Intervention condition membership was negatively associated with Sexual Risk Behavior at baseline (−.23). The intervention condition was positively associated with Positive Parental Bonds at the 18 month follow-up assessment (.23). Parent participation was significantly and negatively associated with Sexual Risk Behavior at baseline (−.23). Parental participation was also significantly associated with less Conflict (−.15) and less Sexual Risk Behavior (−.14) at the follow-up assessment.

### Path Analysis

**Figure 2:**
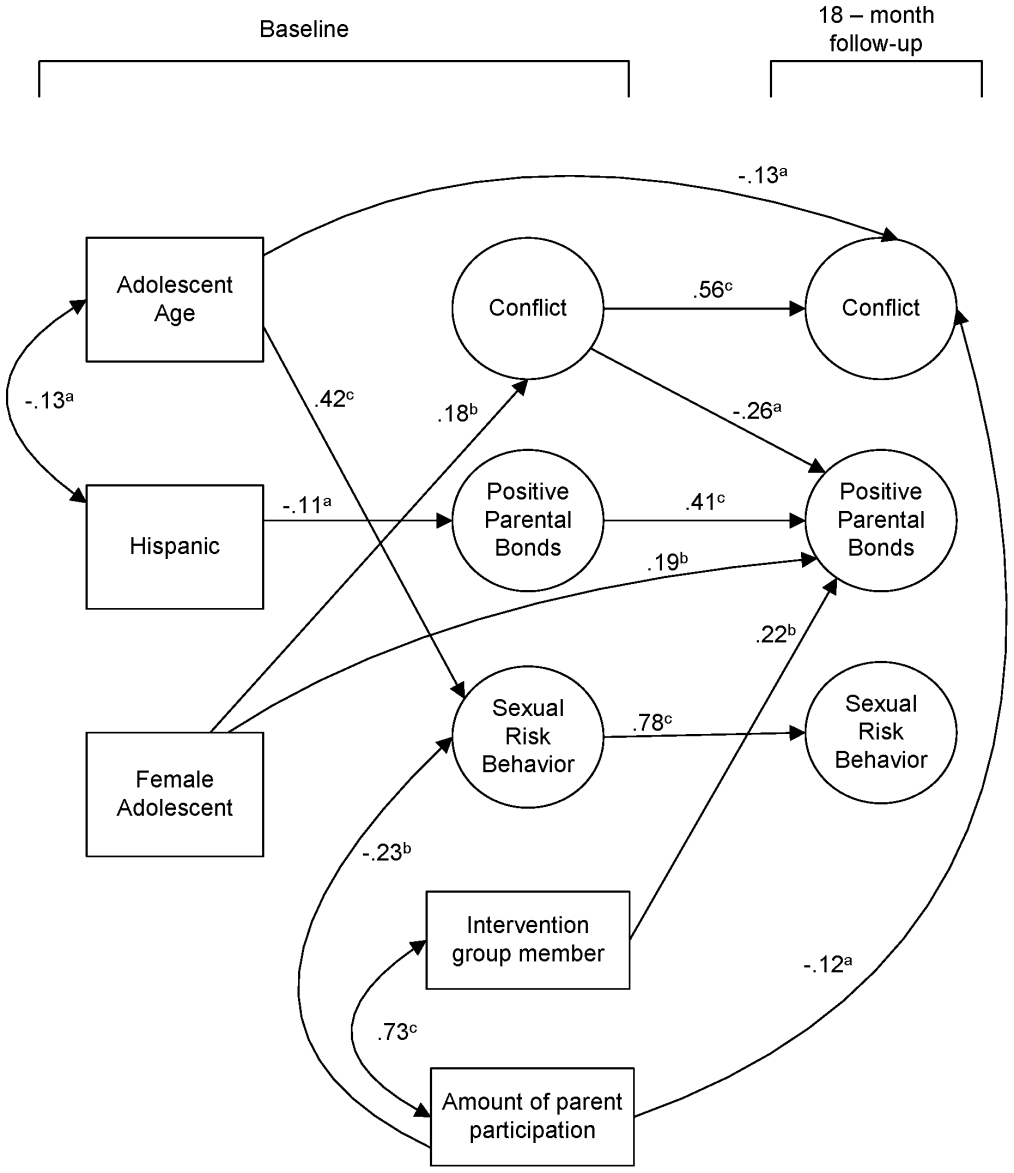
Significant regression paths among latent and measured variables in the structural equation model assessing influences on youth outcomes (*N = *256). Regression coefficients (represented as one-way arrows) are standardized. (a = *p*≤.05, b = *p*≤.01, c = *p*≤.001.).

The final path model was initially tested with the background demographic variables predicting the baseline latent variables. In turn, all of the baseline latent variables predicted the outcome latent variables. After model trimming, the final model is presented in Figure 2. Paths were trimmed gradually following the procedure of MacCallum [51] in which the most minimal non-significant paths and correlations are dropped sequentially, until none remain in the model. Based on suggestions from the LM Test, two paths from the baseline demographic items were added to 18-month outcomes, including a negative path from age and 18-month Conflict (−.13), and between being a Female Adolescent and having Positive Parental Bonds (.19). Fit indexes were very good: S-B χ^2^ = 230.83, 214 *df*; RCFI = .97; RMSEA = .020, CI = .000 to.037.

Not all significant bivariate relationships noted in the CFA remained in the path model, once simultaneous relations among other variables were accounted for. Some relationships that were non-significant in the bivariate analysis were significant in the path analysis. Stability paths were all highly significant. In addition, greater Conflict at baseline predicted lower Positive Parental Bonds at 18-months. Older adolescents reported more Sexual Risk Behavior at baseline. Hispanic youth tended to report lower Parental Bonds at baseline and females reported more Conflict with their MLH at baseline.

Our main focus was assessing the impact of the intervention. As noted in the bivariate correlations, neither gender nor age of the adolescent was significantly associated with intervention condition membership, indicating that randomization had been successful. Assignment to the intervention was significantly associated with having more Positive Parental Bonds at 18 months compared to the standard care condition (.22; *p = *.01). At baseline, the association between Positive Parental Bonds and intervention assignment was negligible. Greater parent participation was associated with having less Conflict at 18 months (−.12, *p = *.05). At baseline, amount of participation was not significantly associated with conflict.

### Indirect effects

Parent participation in the intervention was significantly associated with less Sexual Risk Behavior at 18-months in the bivariate analysis. However, it did not directly predict less Sexual Risk Behavior in the full path model. However, there was a significant indirect effect of parent participation on Sexual Risk Behavior mediated through its substantial association with Sexual Risk Behavior at baseline (standardized indirect effect = −.18, *p*<.05). Parents who participated more fully in the intervention were already influencing their children’s sexual behavior prior to the intervention’s initiation. There also was a significant indirect effect of age on 18-month Sexual Risk Behavior mediated through Sexual Risk Behavior at baseline (.33, *p*<.001).

There were no significant indirect effects on Positive Parental Bonds. There was a significant indirect effect of female gender on Conflict at 18-months (.10, *p*<.05). Girls were more likely to report high Conflict scores at baseline and at 18 months.

## Discussion

In contrast to adolescents of parents living with HIV in New York City a decade earlier, the lifestyles of adolescents in Los Angeles had much less risk in terms of their sex and drug use behaviors, and fewer mental health symptoms [52]. Our previous intent-to-treat analyses indicated that the adapted Project TALC intervention did not influence these outcomes compared to those receiving the standard care intervention [52]. Although there was too little substance use among the adolescents to look at changes in substance use, we were able to examine changes in several critical parent-child relationship domains in the current analysis, including parental bonds, family conflict, and adolescent sexual behaviors.

There were two important direct effects of the intervention on improved adolescent outcomes at 18 months. Youth who were in the intervention condition reported higher levels of parental bonding at 18 months than the standard care condition, and youth whose MLH participated in more intervention sessions reported less family conflict at 18 months. Project TALC appears to improve the quality of family life over time for the adolescents of MLH. Adolescence is often characterized as a time of increased conflict and decreased bonds [53]. However, participation in Project TALC resulted in better relationships over time among MLH and their children, as compared to those in the standard care condition. Note from the means presented in Table 1 that bonds declined over time in both conditions, which is normative among adolescents [53]. However, the intervention was protective in that bonds remained higher among adolescents in the intervention condition.

In Project TALC delivered in New York City, there were significant reductions in sexual risk, substance use, pregnancy, and parenthood among the adolescents of MLH [4]. In the current study, the amount of parental participation was indirectly associated with reductions in sexual risk taking. The reductions in sexual risk were mediated through adolescents’ baseline levels of sexual risk, as would be expected. We can speculate that parents who were more engaged in the program were also parents who monitor their children’s behavior more closely. Parental monitoring has been related to lower rates of sexual behavior [54]. Previously, we found that youth’s participation in Project TALC was associated with reductions in sexual risk [13]. This study finds that youth benefitted not only from their own participation in the intervention, but also benefited by Project TALC participation by their MLH. These intergenerational effects reinforce the findings of the original New York City intervention study conducted in the 1990s [4], [15].

Past behaviors are the best predictors of current behaviors and there are normative developmental patterns for family conflict and bonds during adolescence. As expected, an adolescent’s sense of family conflict at baseline was a strong predictor of family conflict at 18 months. Similarly, initial bonding was related to later bonding, as was adolescents’ sexual risk taking. Those who were more sexually active initially were more likely to be sexually active 18 months later. Higher levels of conflict at baseline was also associated with weaker bonds 18 months later, again, an anticipated finding. Female adolescents, in particular, reported both higher levels of conflict and stronger bonds with their mothers, than male adolescents.

As prior work has demonstrated [15]–[17], [55], parent-adolescent relationships are taxed when mothers are living with HIV. Now that HIV can be considered a chronic illness, the impact of maternal HIV is going to be seen in the persistence of ongoing maternal stress, difficulties in adherence to medical regimens, adversity in coping with daily hassles of symptoms over time, and higher rates of a broad range of diseases (e.g., cancer). Chronic diseases often result in lower quality of life, especially related to the stress associated with these hassles [56]. Project TALC appears to mitigate the stresses. Adolescents perceived less conflict and reported feeling more bonded than their peers facing similar challenges in the control condition.

This study has several limitations. First, our data are self-reports and subject to the biases of self-reported data. Second, attrition was 6% over 18 months; it is possible that the results are sensitive to these lost cases although this rate of attrition is quite modest. Third, while this was an RCT, the sample consisted of MLH seeking services, so we caution readers about generalizing the efficaciousness of this intervention to families of mothers who are not actively seeking care for their HIV. There are unlikely to be selection effects in intervention attendance as 97% of MLH of adolescents attended the intervention. Furthermore, our sample may not be representative of MLH nationally. MLH in Los Angeles engaged in very few risk behaviors themselves, such as injection drug use, and were likely infected by their primary sexual partners [57]. In other parts of the United States, MLH may be more likely to be infected from having multiple sexual partners, or have a history of injecting drug use (e.g., Bronx, New York or Washington D.C.). The adolescents of MLH with long histories of risky lifestyles of using drugs or bartering sex may be at greater risk of negative outcomes due to their modeling of parental behavior (similar to the New York City sample), and the impact of Project TALC may be greater on the young people with this higher risk, as was observed in our earlier study [3].

Our findings support implementing future interventions for MLH that approach HIV as a chronic illness and develop the coping skills and parenting skills that will enable MLH to reduce family conflict and strengthen parent-child bonds. These interventions appear to be efficacious in decreasing some HIV risk behaviors in their adolescent children. Future research should explore the relationship of the best parenting styles (e.g. authoritarian styles within an appropriate racial/ethnic cultural frame) for improving parental bonding and reducing family conflict. This work would contribute to our growing understanding of how best to improve the developmental outcomes of families living with HIV.
